# Diverse radiotherapy fractionation in malignant melanoma: a case report

**DOI:** 10.3389/fonc.2025.1662686

**Published:** 2025-09-26

**Authors:** Jiayi Shen, Yizhi Ge, Puchang Zhang, Han Gao, Lijun Wang

**Affiliations:** Department of Radiotherapy, The Affiliated Cancer Hospital of Nanjing Medical University, Jiangsu Cancer Hospital, Jiangsu Institute of Cancer Research, Nanjing, China

**Keywords:** malignant melanoma, radiotherapy, melanoma of unknown primary, immunotherapy, targeted therapy, case report

## Abstract

Malignant melanoma (MM) is a highly aggressive tumor, with a median overall survival (mOS) of only 8 to 12 months for its metastatic form. However, studies focusing on the efficacy of different radiotherapy (RT) fractionation regimens for MM are limited. Here, we report the case of a 60-year-old male who presented with a one-month history of intermittent abdominal pain and was subsequently diagnosed with MM. Following disease progression on systemic therapy, the patient was treated with different fractionation regimens, including 5 Gy per fraction and 3 Gy per fraction. After the failure of immunotherapy, RT effectively controlled the tumor burden. Notably, the patient received different doses of RT and achieved different outcomes. This case report demonstrates that RT could serve as a viable option for patients who have developed resistance to immunotherapy and low-dose RT may enhance tumor immune response when combined with immunotherapy.

## 1 Introduction

Malignant melanoma (MM) is s one of the most metastatic human cancers that can arise in the skin, mucous membranes, uvea, and leptomeninges (1). Melanoma of unknown primary (MUP) is defined as metastatic melanoma without a detectable primary lesion, typically found in lymph nodes, subcutaneous tissues, or other distant sites. MUP has a relatively low incidence, accounting for 3-4% of all melanoma cases (2–4). According to the American Joint Committee on Cancer (AJCC) 8^th^ edition staging manual, MUP presenting in lymph nodes or subcutaneous tissue is classified as stage III disease, in contrast, stage IV disease is characterized by distant metastases, including visceral metastases (5). Surgical resection remains the primary treatment for melanoma but is only effective for pre-stage IV disease with minimal regional metastasis (6, 7). For unresectable metastatic melanoma, systemic therapies, particularly immunotherapy and targeted therapy, have become the mainstay of treatment (8, 9). Although melanoma is often radioresistant, radiotherapy remains useful for unresectable or recurrent cases (7).

In this report, we describe a patient with MUP who received multiple courses of radiotherapy (RT). We observed that the irradiated lesions remained stable, with some demonstrating a partial response (PR).

## 2 Case presentation

### 2.1 Patient

On March 14, 2024, a 60-year-old male presented with abdominal pain. A computed tomography (CT) scan revealed multiple soft-tissue nodules in the abdominopelvic cavity, thoracic cavity, and retroperitoneal space (With a total of six lesions measuring greater than 1 cm, and the largest measuring 9.51 × 4.30 cm), along with enlarged lymph nodes in the anterior mediastinum, bilateral phrenic-diaphragmatic angles, lower esophagus, hepatic hilum, perigastric space, and retroperitoneum. Additionally, inflammatory changes were noted in the left ethmoid sinus. Three days later, the patient underwent abdominal paracentesis. The pathological results showed, microscopically, that round and oval cells were densely arranged in sheets, constituting a tumor lesion. Immunohistochemical (IHC) staining was positive for Ki-67 (20%), CD99, S-100, Vimentin, HMB-45, Melan-A, and SOX10, while being negative for SMA. Genetic testing revealed CDK4 amplification but no mutation in BRAF, NRAS, KIT, and no fusion in NTRK1/2/3 or ROS1(**Table 1**).

**Table 1:** Summary of immunohistochemistry and genetic testing results.

IHC	
Positive	Ki-67(20%), CD99, S-100, Vim, HMB45, Melan A and Sox10
Negative	AE1/AE3, P40, CK7, TTF-1, CD3, CD20, CD45LCA, CD30, EBER, TdT, WT-1, NKX2-2, Desmin, CD34, SMA
Genetic test	
BRAF	not mutated
NRAS	not mutated
KIT	not mutated
NTRK1/2/3	not fused
ROS1	not fused
CDK4	amplificated

The patient was diagnosed with stage IV MUP according to the 8th edition of the American Joint Committee on Cancer (AJCC) cutaneous melanoma staging system. He initially received two cycles of chemotherapy with albumin-bound paclitaxel (300 mg) and carboplatin (600 mg). However, disease progression was observed. Subsequently, he was treated with toripalimab (240 mg) and apatinib (250 mg). After three cycles, he achieved stable disease (SD). However, after completion of seven cycles, a repeat CT revealed further disease progression, with the largest lesion increasing in diameter from 10.2 cm to 14 cm.

**Figure 1:**
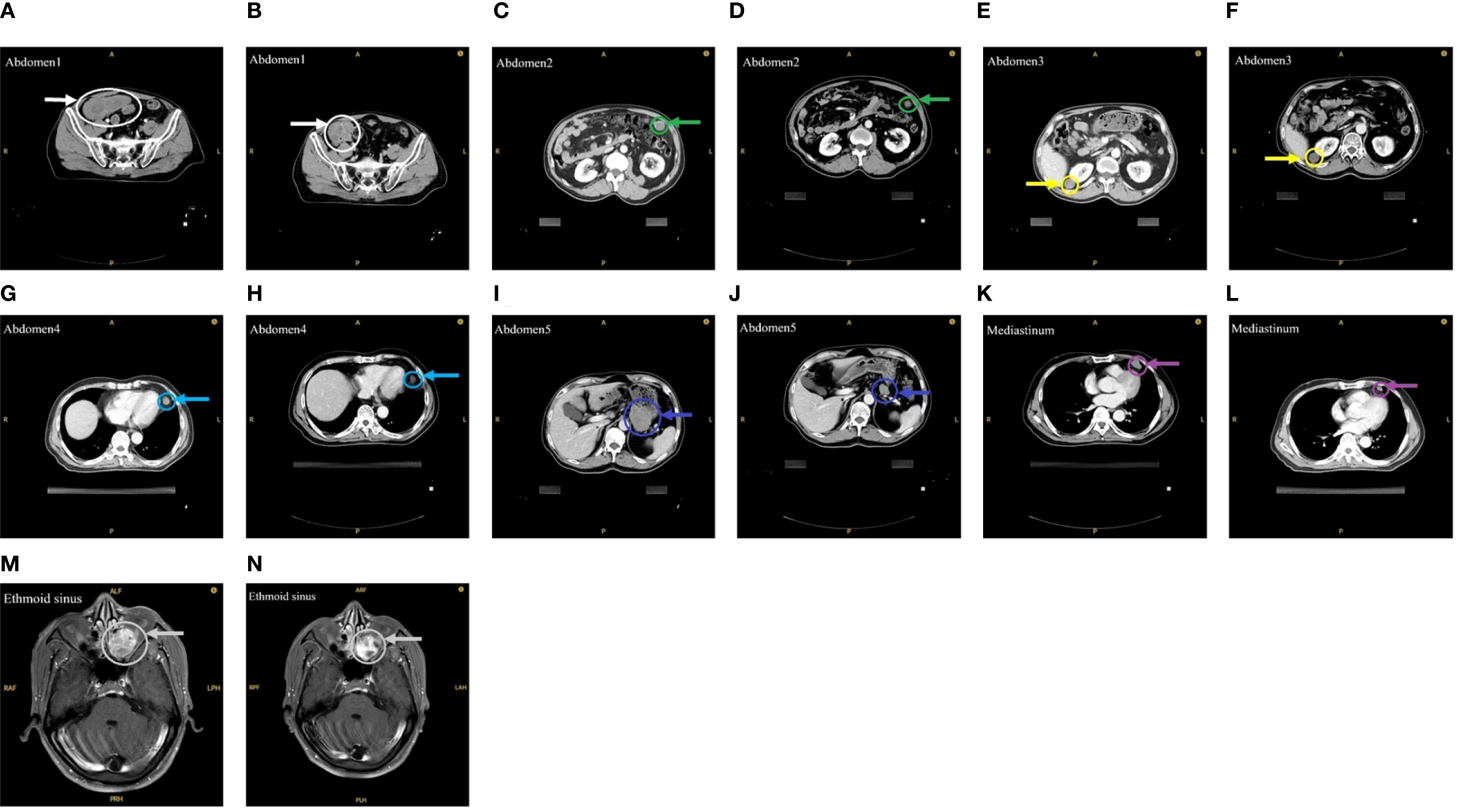
Imaging before and after RT. **(A)** Before the first RT. **(B)** After the first RT. **(C, E, G, I)** Before the second RT. **(D, F, H, J)** After the second RT. **(K)** Before the third RT. **(L)** After the third RT. **(M)** Before the fourth RT. **(N)** After the fourth RT.

On October 28, 2024, the patient commenced stereotactic body radiation therapy (SBRT), with the largest abdominal lesion receiving a total dose of 25 Gy in 5 fractions (Abdomen1: 25 Gy in 5 fractions). Subsequently, the patient commenced a three-week cycle of combination therapy with toripalimab and apatinib on November 11, 2024. A follow-up CT scan one month later demonstrated PR in the irradiated lesion. (**Figures 1A, B**). Two weeks later, he received further RT for the remaining larger abdominal lesions (Abdomen2/3/4: 25 Gy in 5 fractions). To minimize gastrointestinal toxicity, a lesion near the stomach was treated with a lower-dose regimen (Abdomen5: 18 Gy in 6 fractions).

One month after this course of RT, evaluation showed PR in the low-dose field and SD in the high-dose fields (**Figures 1C-J**). Subsequently, the mediastinal lesions were irradiated (Mediastinum: 18 Gy in 6 fractions). Three weeks later, imaging showed regression of the mediastinal lesions (**Figures 1K, L**). During this period, the patient developed nasal bleeding, and magnetic resonance imaging (MRI) revealed a metastasis in the ethmoid sinus. The same radiation dose was administered to this site (Ethmoid sinus: 18 Gy in 6 fractions). A follow-up MRI one month later showed regression of the ethmoid sinus lesion in the (**Figures 1M, N**). After RT, overall tumor burden markedly decreased (**Table 2**). Among the 7 lesions, 3 achieved PR and 4 showed SD. Notably, the 4 lesions treated with high-dose irradiation exhibited an average reduction of 32.27%, while the 3 lesions receiving low-dose irradiation demonstrated a more pronounced average shrinkage of 66.66%.

**Table 2:** Tumor volumes before and after RT.

Tumor lesion	Fractionation regimen (Gy/Gy/F)	Volume before RT (cm^3^)	Volume after RT (cm^3^)	Volume reduction (%)
Abdomen1	25/5/5	281.53	48.00	82.95
Abdomen2	25/5/5	5.69	5.23	8.08
Abdomen3	25/5/5	4.66	3.84	17.60
Abdomen4	25/5/5	1.86	1.48	20.43
Abdomen5	18/3/6	52.35	4.82	90.79
Mediastinum	18/3/6	7.09	0.79	88.86
Ethmoid sinus	18/3/6	10.48	8.35	20.32

Tumor volumes were estimated using the formula V = 0.5 × L × W², where L is the longest diameter and W is the perpendicular short diameter. This method is a rough approximation with inherent inaccuracies and was not used for primary response assessment.

**Figure 2:**
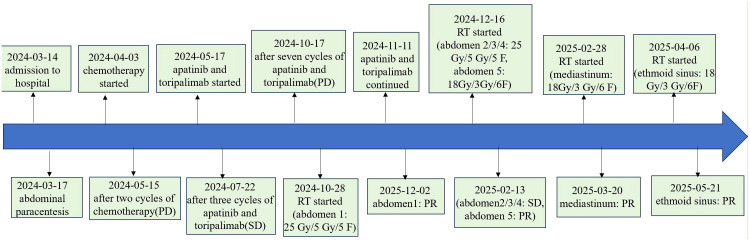
A timeline of the treatment course.

As of May 2025, with over one month elapsed since the final RT session, all irradiated lesions in this patient have maintained PR or SD status. The patient’s Eastern Cooperative Oncology Group (ECOG) performance status was 1, with minimal symptom burden including only mild fatigue. Treatment-related toxicities were limited to grade 1 radiation dermatitis, which showed improvement with symptomatic management. A timeline of the treatment course is provided in **Figure 2**.

### 2.2 Radiotherapy

All treatments were delivered using a Varian TrueBeam linear accelerator with 6 MV photon energy, where the dose rate was 1.2 Gy/min for high-dose regimens and 0.6 Gy/min for low-dose regimens. SBRT was used for high-dose irradiation (25 Gy in 5 fractions), while conventional fractionation was employed for low-dose irradiation (18Gy in 6 fractions). RT was administered once daily, five consecutive days per week (Monday to Friday). Response to RT was monitored via serial CT and MRI, with lesion dimensions measured according to RECIST 1.1 criteria. Treatment details, including target volume delineations, and plan evaluation, are provided in **Supplementary Materials**.

## 3 Discussion

Melanoma is a highly aggressive malignancy with a rapid progression and poor prognosis, causing approximately 55,000 deaths worldwide annually (10). The diagnosis can be aided by IHC and genetic testing. Common positive IHC markers include S-100, SOX-10 and Melan-A (11), while frequent genetic alterations include BRAF and NRAS mutations (11–15). In this case, the patient’s non-specific clinical presentation and inconclusive imaging findings complicated the initial diagnosis. However, the diagnosis of MM was confirmed through IHC (positive for S-100, SOX10, HMB45, and Melan-A) and histopathological examination. With no prior history of melanoma and no detectable primary lesion upon comprehensive assessment, the patient was diagnosed with melanoma of unknown primary MUP. Notably, inflammatory changes in the left ethmoid sinus were noted at the patient’s initial admission. Following treatment, a lesion developed in the ethmoid sinus. Given the spontaneous regression potential of MM, whether the lesion represented a primary or metastatic focus remained unclear. The patient’s initial treatment with chemotherapy was ineffective.

In recent years, immune checkpoint inhibitors (ICIs) and targeted therapies have significantly improved survival outcomes for patients with advanced melanoma (16). High tumor mutational burden (TMB) is a biomarker for better response to ICIs (17), making ICI-based therapy a cornerstone for metastatic MM (18). Combining anti-angiogenic agents with PD-1 inhibitors can enhance anti-tumor activity and mitigate resistance (19). For instance, toripalimab plus axitinib showed a 48.3% objective response rate (ORR) in advanced mucosal melanoma (20), and lenvatinib plus pembrolizumab provided durable responses in patients with advanced MM who had progressed on prior anti-PD-1 therapy (21). In our case, the patient achieved SD with a PD-1 inhibitor plus an anti-angiogenic drug, suggesting initial efficacy, but eventually developed resistance after seven cycles (22).

Compared to cutaneous melanoma, other melanoma subtypes have fewer BRAF mutations and more frequent KIT mutations (11, 23). This patient had neither, making him ineligible for BRAF or KIT inhibitors. CDK4 gene amplification, an important genetic feature in MM (24), can be targeted (25), but clinical trials of the CDK4 inhibitor abemaciclib have shown low ORRs (0-3.8%) in advanced MM patients (26, 27), and no CDK4 inhibitor has been approved for melanoma treatment to date. Therefore, CDK4 inhibitors were not administered, but clinical trials are needed to clarify their role.

To date, the patient has received four courses of RT. MM is traditionally considered radioresistant, partly due to a low α/β ratio and a high capacity for sublethal damage repair under conventional fractionation (7, 28). Under conventional fractionation, MM has a strong ability to repair sublethal damage, and the cytotoxic effect of conventional fractionation may be offset by efficient sublethal damage repair in melanoma cells (7). Early studies on melanoma showed a complete response rate of 82% (range 67-92%) for patients receiving >4 Gy/F and only 36% (range 21-46%) for <4 Gy/F (25–29). These findings have led to the widespread adoption of hypofractionated radiotherapy for melanoma treatment. The most commonly used regimen delivers 30 Gy in 5 fractions of 6 Gy each, administered twice weekly, with comparable efficacy observed across both cutaneous and mucosal subtypes (30). However, RTOG8305 was a prospective clinical study that included 137 patients with MM, with one group of patients treated with high-dose RT (32 Gy in 4 fractions) and one group treated with low-dose RT (50Gy in 20 fractions) (31). There was no significant difference in tumor regression or local failure rates between the two groups, with an increase in grade 4 toxicity in the high-dose group (31). TROG96-06, a randomized prospective clinical study, reached the same conclusions using the same dose (32). Currently, there is no consensus on the mode and dose of segmentation for MM.

More recently, the combination of ICI and RT has shown promise, even in patients who have failed prior anti-PD-1 therapy (33–35). Preclinical evidence indicates that RT enhances antitumor immunity through multiple mechanisms, such as promoting dendritic cell-mediated antigen presentation, increasing the release of immune-stimulatory mediators, and fostering a pro-inflammatory tumor microenvironment (TME) (36). Funck-Brentano et al. analyzed 26 consecutive patients with advanced melanoma who progressed on ICI and reported that 10 patients (38%) achieved a complete response (CR) or partial response (PR) following combined ICI and hypofractionated RT (37). However, the immunostimulatory effects of RT are influenced by dose and fractionation. High-dose irradiation can induce immunogenic tumor cell death and release tumor-specific antigens (38), while low-dose irradiation may enhance the activation and stimulation of immune cells as well as modulate the stromal microenvironment, thereby potentiating the efficacy of immunotherapy (39, 40). A phase I trial of ipilimumab and SBRT suggested that lower radiation doses (e.g., 24 Gy in 3 fractions) might be more synergistic with immunotherapy, as higher doses could have an antagonistic effect on the immune response (41–43). One case report described a patient with metastatic vaginal mucosal melanoma who was treated with combined immunotherapy and RT. The patient received varying RT doses: high-dose (30 Gy in 5 fractions) to two liver metastases, low-dose (5 Gy in 5 fractions) to another liver lesion, and low-dose (6 Gy in 6 fractions) to a right inguinal lesion, followed by continued immunotherapy. At 24-month follow-up, all irradiated lesions achieved complete response (CR) (44). Another recent case reported local improvement with low-dose scatter radiation (0.9-1.8 Gy) in a patient with stage IV MUP (45). In the present case, we hypothesize that low-dose irradiation may more effectively induce immunogenic cell death and facilitate tumor antigen release. Nevertheless, no significant abscopal effect was observed throughout the treatment course.

In our report, we observed that the lesion treated with a lower dose (18Gy in 6 fractions) demonstrated superior tumor burden reduction compared to those treated with the higher-dose fractions. This finding appears inconsistent with some literature but highlights a critical point: the radiosensitivity of lesions can be heterogeneous, even within the same patient. Studies have revealed significant heterogeneity in RT responses, which arises from complex interactions between radiation dose and TME (46, 47). Using B78 melanoma and MyC-CaP prostate cancer mouse models, Jagodinsky et al. demonstrated that varying radiation doses can induce distinct biological and treatment outcomes even within a single tumor (48).

Several limitations inherent to this case report should be acknowledged. First, the absence of correlative data at the molecular level and immunological parameters precludes validation of the proposed mechanistic hypotheses. Second, the short follow-up period limits the assessment of long-term local control and overall survival outcome. Furthermore, conclusions are constrained by the nature of a single-case report. Finally, the primary site of origin remains undetermined throughout the treatment course, and it is unclear whether the ethmoid sinus lesion represents a primary tumor or a metastatic deposit.

## 4 Conclusion

In summary, we present a case of MUP with multiple metastases where the diagnosis was confirmed by pathological and immunohistochemical analysis. RT provided effective local control after the patient developed resistance to systemic immunotherapy. This case suggests that RT is a viable option following the development of immune resistance and that lower-dose fractionation may, in some instances, elicit a superior anti-tumor response. However, determining the optimal timing, dose, and fractionation schedule for RT, especially in combination with immunotherapy, remains a significant challenge. Further research is imperative to develop individualized and optimized treatment strategies for patients with MM.

## Data Availability

The original contributions presented in the study are included in the article/**Supplementary Material**. Further inquiries can be directed to the corresponding author.
