# Supplementary material for: Diverse radiotherapy fractionation in malignant melanoma: a case report
Source: Front Oncol. 2025 Sep 26;15:1662686. doi: 10.3389/fonc.2025.1662686 (PMC12510920; doi:10.3389/fonc.2025.1662686)
Supplement: Supplementary file 1 [file DataSheet1.docx]

***Supplementary Material***

1. **Supplementary Figures**


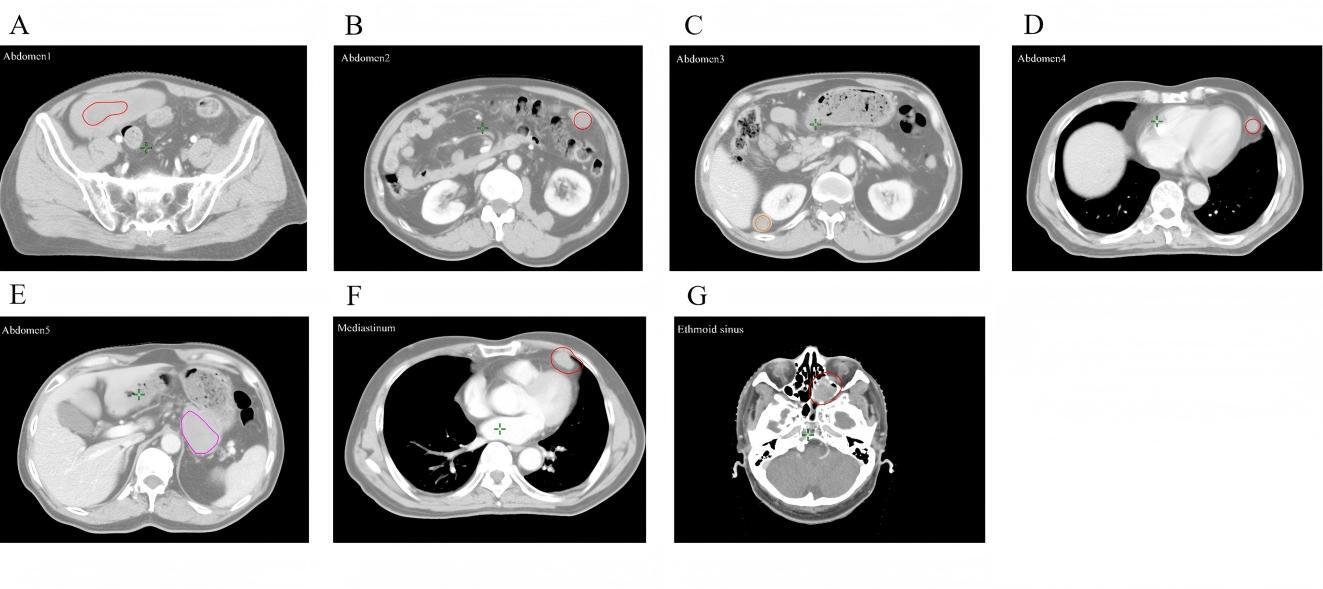


**Figure 1.** CT guided gross tumor volume (GTV) delineation. (A) The first RT. (B/C/D/E) The second RT. (F) The third RT. (G) The fourth RT.


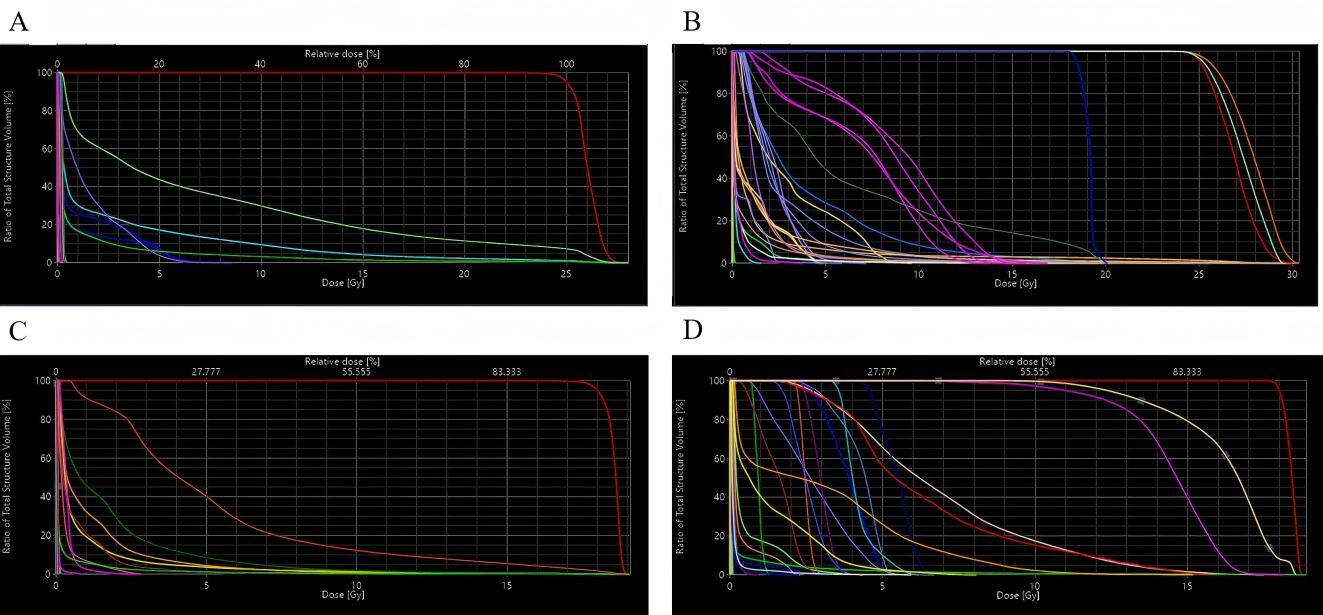


**Figure 2.** DVHs for RT plans. (A) First RT: GTV of abdomen1 (red; D_95%_ = 24.9 Gy). (B) Second RT: GTVs of abdomen2 (red; D_95%_ = 25.1 Gy), abdomen3 (orange; D_95%_ = 25.5 Gy), abdomen4 (green; D_95%_ = 25.2 Gy), and abdomen5 (bule; D_95%_ = 18.4 Gy). (C) Third RT: GTV of mediastinum (red; D_95%_ = 18.0 Gy). (D) Fourth RT: GTV of ethmoid sinus (red; D_95%_ = 18.0 Gy).


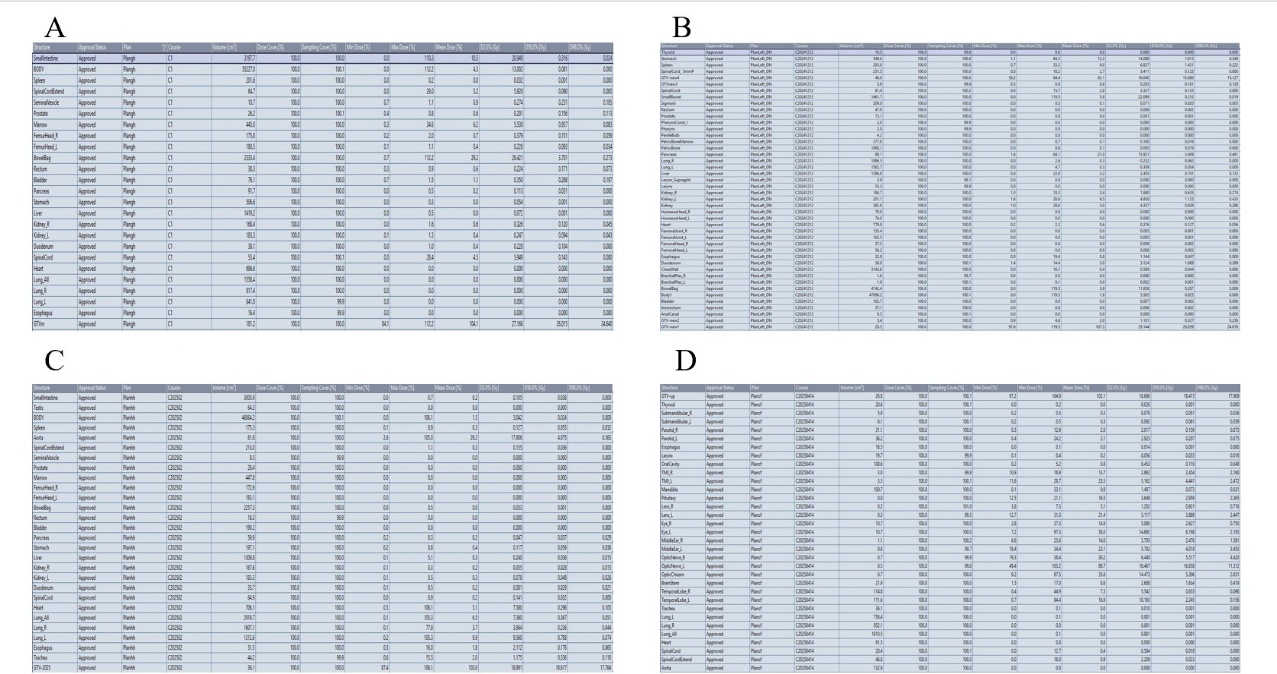


Figure 3. Actual radiation doses received by organs at risk (OARs). (A) The first RT. (B) The second RT. (C) The third RT. (D) The fourth RT.


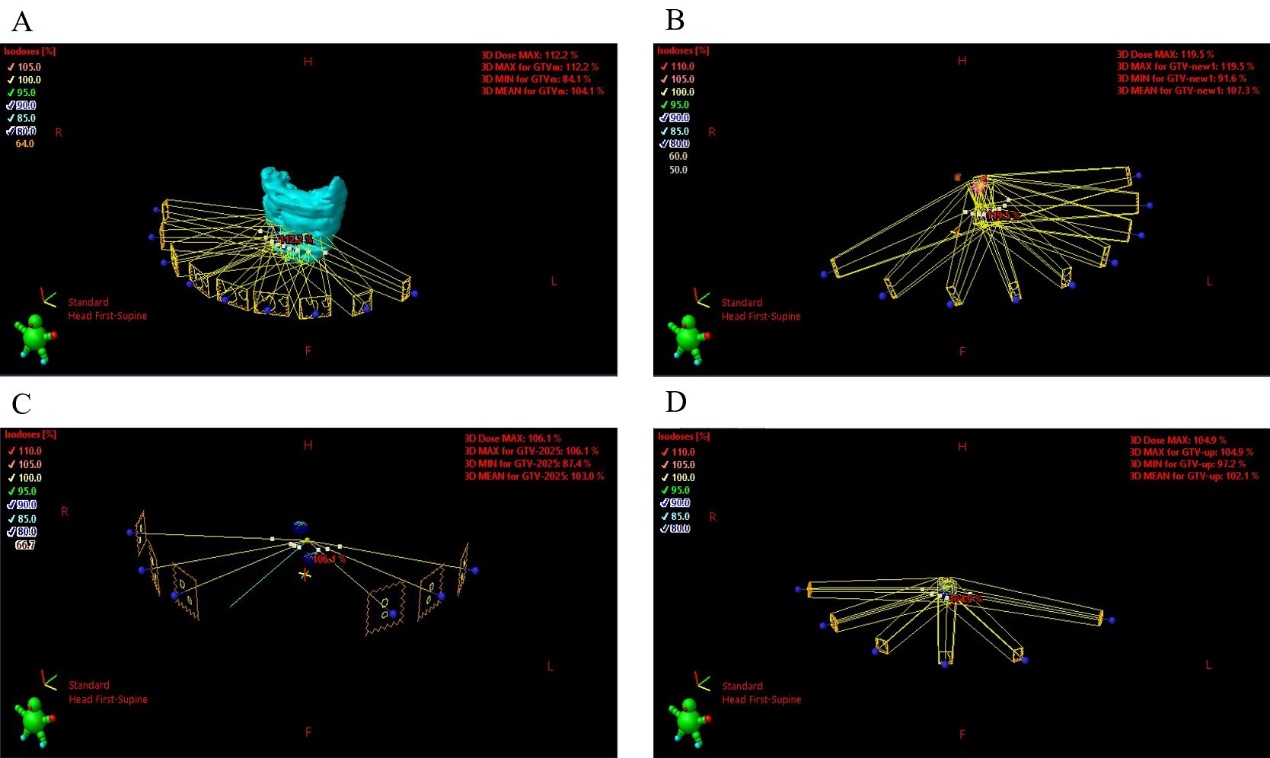


Figure 4. Beam arrangement in radiation therapy. (A) The first RT. (B) The second RT. (C) The third RT. (D) The fourth RT.

1. **Organ at Risk Constraints**
2. **The first RT**

- Small bowel & colon: Dmax < 52 Gy
- Bladder: V50 < 50%
- Femoral heads: V50 < 50%
- Perineum: V20 < 50%
- Bone marrow: V20 < 70%, V30 < 50%, V40 < 30%

1. **The second RT**

- Heart: V32 < 15 cm³, Dmax < 38 Gy
- Stomach: V26.5 < 5 cm³, Dmax < 35 Gy
- Intestine (small/large bowel): V32.5 < 20 cm³, Dmax < 52.5 Gy

1. **The third RT**

- Lungs: V5 < 60%, V20 < 30%, V30 < 20%
- Heart: V40 < 30%, V30 < 40%

1. **The fourth RT**

- Brainstem: Dmax ≤ 54 Gy
- Temporal Lobes (L/R): Dmax ≤ 54 Gy
- Spinal Cord: Dmax ≤ 45 Gy
- Eyes (L/R): D50% ≤ 35 Gy
- Lens: D1% ≤ 6 Gy
- Parotids Glands (L/R): D50% ≤ 20 Gy
- Submandibular Glands (L/R): D50% ≤ 30 Gy
- Thyroid: D50% ≤ 45 Gy
- Larynx: Dmean ≤ 35 Gy
- Oral Cavity: Dmean ≤ 40 Gy
